# Research Priorities in Prehabilitation for Patients Undergoing Cancer Surgery: An International Delphi Study

**DOI:** 10.1245/s10434-023-14192-x

**Published:** 2023-08-24

**Authors:** Pratik Raichurkar, Linda Denehy, Michael Solomon, Cherry Koh, Neil Pillinger, Sophie Hogan, Kate McBride, Sharon Carey, Jenna Bartyn, Nicholas Hirst, Daniel Steffens, Prehabilitation Expert Collaborative, Jonathan Allen, Kevin Ancog, Eva Angenete, Nabila Ansari, Fabio Ausania, Anna Beaumont, Christian Beilstein, Frederik Berrevoet, Ianthe Boden, Kimberley Bostock, Janine Bothe, Birgitte Brandstrup, Louise Brennan, Kilian Brown, Sorrel Burden, Crystal Burgess, Elaine Burns, Francesco Carli, Vinicius Cavalheri, Wim Ceelen, Tyler Chesney, David Clark, Kari Clifford, Kelcie Cole, Thomas Collyer, Rob Copeland, Roland Croner, Jess Crowe, Ian Daniels, Gerard Danjoux, June Davis, Caitlin Davis, Mayke de Klerk, Tina Decorte, Jan Willem Dekker, Andreas Denys, Liesbeth Desender, Pieter Dries, Declan Dunne, Lara Edbrooke, Linda Edgar, Sabry Eissa, Dominique Engel, Martyn Evans, Rhonda Farrell, Alice Finch, Aisling Fleury, Patrice Forget, Nader Francis, Frank Frizelle, Walter Frontera, Karen Geboes, Hugh Giddings, Chris Gillespie, Chelsia Gillis, Olivier Glehen, Varsha Gorey, Catherine Granger, Diana Greenfield, Ben Griffiths, Chloe Grimmett, Claire Hackett, Travis Hall, Julie Hallet, Craig Harris, Sophie Hatcher, Lizza Hendriks, Mendy Hermans, Carl Ilyas, Hilmy Ismail, John Jenkins, Wilson Jiang, Charlotte Johnstone, Andreas Karakatsanis, Sascha Karunaratne, Simarjit Kaur, Michael Kelly, Joost Klaase, Dorian Kršul, Scott Leslie, Jenelle Loeliger, Marie-Louise Lydrup, Andrea Maier, Piotr Major, Preet Makker, Christopher Mantyh, Stuart McCluskey, Laura McGarrity, Jayson Moloney, Isacco Montroni, Brendan Moran, Paul Morris, Susan Moug, Rajeswari Ms, Sandra Murdoch, Anna Myers, Kheng-Seong Ng, Per J. Nilsson, Peter Noordzij, Mike O’Connor, Gianluca Pellino, Shannon Philp, Marc Pocard, Zudin Puthucheary, Emma Putrus, Aaron Quyn, Thomas Read, William Ricketts, Bernhard Riedel, Harm Rutten, Charissa Sabajo, Rawand Salihi, Tarik Sammour, Charbel Sandroussi, Daniel Santa Mina, Stefan Saric, Raquel Sebio, Doruk Seyfi, Favil Singh, Gerrit Slooter, Neil Smart, Lissa Spencer, Paul Sutton, Hao Ern Tan, David Ten Cate, Akif Turna, Elke Van Daele, Adinda van den Berg, Charlotte van Kessel, Gabrielle van Ramshorst, Emiel Verdaasdonk, Jennifer Vu, Chris Wakeman, Malcolm West, James Wheeler, Duminda Wijeysundera, Hideaki Yano

**Affiliations:** 1 Surgical Outcomes Research Centre (SOuRCe), Royal Prince Alfred Hospital (RPAH), Sydney, NSW Australia; 2 Department of Health Services Research: Allied Health, Peter MacCallum Cancer Centre, Melbourne, VIC Australia; 3 Department of Physiotherapy, Faculty of Medicine Dentistry and Health Sciences, The University of Melbourne, Melbourne, VIC Australia; 4 Institute of Academic Surgery (IAS), Royal Prince Alfred Hospital (RPAH), Sydney, NSW Australia; 5 Faculty of Medicine and Health, Central Clinical School, The University of Sydney, Sydney, NSW Australia; 6 Colorectal Department, Royal Prince Alfred Hospital (RPAH), Sydney, NSW Australia; 7 Department of Anaesthetics, Royal Prince Alfred Hospital (RPAH), Sydney, NSW Australia; 8 Nutrition and Dietetics Department, Royal Prince Alfred Hospital (RPAH), Sydney, NSW Australia

**Keywords:** Cancer, Delphi, Prehabilitation, Perioperative care, Research priorities, Surgery

## Abstract

**Background:**

Recently, the number of prehabilitation trials has increased significantly. The identification of key research priorities is vital in guiding future research directions. Thus, the aim of this collaborative study was to define key research priorities in prehabilitation for patients undergoing cancer surgery.

**Methods:**

The Delphi methodology was implemented over three rounds of surveys distributed to prehabilitation experts from across multiple specialties, tumour streams and countries via a secure online platform. In the first round, participants were asked to provide baseline demographics and to identify five top prehabilitation research priorities. In successive rounds, participants were asked to rank research priorities on a 5-point Likert scale. Consensus was considered if > 70% of participants indicated agreement on each research priority.

**Results:**

A total of 165 prehabilitation experts participated, including medical doctors, physiotherapists, dieticians, nurses, and academics across four continents. The first round identified 446 research priorities, collated within 75 unique research questions. Over two successive rounds, a list of 10 research priorities reached international consensus of importance. These included the efficacy of prehabilitation on varied postoperative outcomes, benefit to specific patient groups, ideal programme composition, cost efficacy, enhancing compliance and adherence, effect during neoadjuvant therapies, and modes of delivery.

**Conclusions:**

This collaborative international study identified the top 10 research priorities in prehabilitation for patients undergoing cancer surgery. The identified priorities inform research strategies, provide future directions for prehabilitation research, support resource allocation and enhance the prehabilitation evidence base in cancer patients undergoing surgery.

Prehabilitation in recent years has gained importance as an approach to enhance preoperative health and consequently improve postoperative outcomes in surgical patients.^1–3^ Prehabilitation in cancer care occurs in the time between diagnosis and surgical management, identifying impairments and delivering targeted interventions to improve health with the goal of preventing adverse effects of treatment.^4,5^ Preoperative interventions encompass optimisation of physical, nutritional, and/or psychosocial health.^6,7^ Annually, over 9 million individuals worldwide undergo cancer surgery, and this number is predicted to increase.^8^ These patients are often quite vulnerable as high-value surgery may carry complications, psychological burden and impacts to quality of life. There is growing evidence that prehabilitation may improve preoperative functional capacity, promote recovery, reduce complications, and reduce healthcare costs.^1,2,7,9^

Currently, most of the literature in this field is from small, underpowered trials, employing a heterogenous mix of interventions and protocols.^10^ While recent trials and systematic reviews demonstrate the significant benefits of prehabilitation in improving preoperative functional capacity and postoperative outcomes, including in-hospital complications and length of hospital stay, this field remains in its infancy. A clearer focus and identification of key research priorities is needed to inform the development of future trials and contribute to the current knowledge base.^6,11^ In addition, this information will generate new opportunities for investigators and allow for better direction of critical resources to support needed areas of research.

The Delphi methodology is a validated and well-established approach to systematically collate ideas and move towards consensus on important research topics among key stakeholders. This comprehensive methodology has been used widely to identify research priorities and core outcomes for other conditions and populations.^12–14^ Traditionally, an open-ended question is proposed, followed by sequential anonymous survey rounds to achieve consensus across a panel of experts.^15^

The aim of this international collaborative study was to determine the top prehabilitation research priorities for patients undergoing cancer surgery; based on the consensus opinion of a multidisciplinary group of prehabilitation experts.

## Methods

### Study Design and Setting

This Delphi study was co-ordinated by the Surgical Outcomes Research Centre (SOuRCe) in conjunction with the Institute of Academic Surgery (IAS) at Royal Prince Alfred Hospital, Sydney, New South Wales, Australia. This manuscript followed recommendations from Conducting and REporting of DElphi Studies (CREDES) and Core Outcome Measures in Effectiveness Trials (COMET initiative) statements on executing Delphi studies.^15–17^ A three-round Delphi survey was conducted using secure online distribution software, Research Electronic Data Capture (REDCap), between May and November 2022. Ethical approval was obtained from the Sydney Local Health District Ethics Review Committee on 2 November 2021 (approval number X21-0361/ETH11714).

### Participants

International multidisciplinary experts in prehabilitation included clinicians and/or academics involved in research surrounding prehabilitation in the areas of medicine, nursing, physiotherapy, dietetics, and psychology. Experts were identified via the following approaches.

1. Literature identified in a comprehensive prehabilitation systematic review.^18^ Corresponding authors of the identified published literature who had provided an email address were contacted for participation.
2. Editorial boards of relevant international peer-reviewed journals were identified via Journal/Author Name Estimator software (https://jane.biosemantics.org/) and contacted via email.
3. Websites of international academic institutions in prehabilitation were identified and key individuals were invited to participate.
4. Investigators overseeing this study utilised their research network to invite additional participants.
5. Invited participants were also encouraged to forward the study invitations to their relevant networks.

Overall, approximately 650 potential participants were invited; responders to the initial invitation and information guide were included in the study.

### Delphi Methodology

The Delphi methodology encompassed three rounds of surveys. An open-ended statement was provided and expert responses were refined through consensus across two subsequent rounds. Reminder emails were sent to the prehabilitation experts who had not responded to the initial email (10 and 20 days from the initial email). The results of each round were collated after 30 days.

### First Round

The first round of the study consisted of two sections. The first section collected baseline demographic information, (i.e., age, sex, professional position, highest level of education, country of residency, main cancer population treated, number of cancer procedures performed, prehabilitation standard of care, number of years of experience in prehabilitation, number of publications, main prehabilitation area of interest). The second section included the following open-ended statement “list up to five research priorities in prehabilitation research for patients undergoing cancer surgery”. Participant responses were categorised into common themes (e.g., exercise, outcome measurements) and contributing responses within these were aggregated into a single research priority statement. The number of constituent responses was recorded. Responses were aggregated independently by four prehabilitation experts, and disagreements were discussed until consensus was reached.

### Second Round

In the second round, aggregated research priorities that had been identified more than three times in round one were redistributed to participants. These priorities contained a brief description capturing the responses that made up each priority. In this round, participants were asked to rate each of the collated priorities on a 5-item Likert scale (1 = very high; 2 = high; 3 = moderate; 4 = low; or 5 = very low research priority). The priorities were presented in a randomised order to avoid bias. Research priorities in which 70% of participants had rated as very high or high research priorities were collated for the third survey round. This criterion was used to define consensus for this study. The remaining research priorities were discarded. In this round, participants were also asked if there were any priorities missing from the list or if adjustments should be made to the collated priorities.

### Third Round

In the third round, the focused list of research priorities was presented to participants in a randomised order. Participants were asked to rate these on a 5-item Likert scale (1 = very high; 2 = high; 3 = moderate; 4 = low; or 5 = very low research priority). Research priorities that reached consensus of at least 70% of participants rating them as very high or high priorities were used to form the final top research priorities in prehabilitation. Open-ended feedback was collected from participants to identify any missing research priorities or adjustments required to existing collated priorities.

### Statistical Analysis

Descriptive statistics were employed to summarise the characteristics of the participants. For round one, qualitative analysis was used to collate responses into sufficiently similar research priorities, and these were further described through frequency (percentage). For the second and third rounds, research priorities were described as frequency (percentage). Additionally, median scores of the 5-item Likert scale (1 = very high; 2 = high; 3 = moderate; 4 = low; or 5 = very low research priority) were calculated. The final prehabilitation research priorities that reached consensus (> 70% rated as high or very high research priority) were presented.

## Results

Overall, 650 prehabilitation experts were identified and contacted for participation in the study. Of these, a total of 165 (25%) responded to the invitation to participate and completed at least one round of the study. A total of 142 (86%) experts participated in the first round, 126 (76%) participated in the second round and 118 (72%) participated in the third round.

The demographic information of the included participants is summarised in Table 1. Participants had an average of 6.18 years of experience in prehabilitation. The majority of participants had a medical background (58%); however, overall, the sample encompassed multidisciplinary experts, including physiotherapists, dieticians, nurses, psychologists, and academics.

**Table 1:** Baseline demographics of the included participants [*N *= 165]

Characteristics	Participants^a,b^
*Profession*	
Medical	96 (58)
Surgery	60 (36)
Anaesthesiology	17 (10)
Physiotherapy	17 (10)
Dietetics	15 (9)
Psychology	1 (1)
Nursing	8 (5)
Academic	7 (4)
Not specified	20 (12)
*Sex*	
Male	84 (51)
Female	61 (37)
Not specified	20 (12)
*Age, years*	
<30	12 (7)
31–40	39 (24)
41–50	55 (33)
51–60	27 (16)
61–70	10 (6)
>70	2 (1)
Not specified	20 (12)
*Highest level of education*	
Bachelors	17 (10)
Masters	33 (20)
Doctoral degree	42 (25)
Professional doctorate	47 (28)
Not specified	26 (16)
*Years of experience in prehabilitation*	6.18 ± 5.54
*cancer populations treated*	
Colorectal	122
Pancreatic	59
Liver	57
Gastric	59
Oesophageal	62
Urological	52
Lung	57
Other	36
Not specified	20
*Continent of Practice*	
Australia/Oceania	55 (33)
Asia	4 (2)
Europe	73 (44)
North America	13 (8)
Not specified	20 (12)

^a^Data are expressed as frequency (percentage) or mean ± standard deviation

^b^Values are reported rounded to the nearest whole number unless otherwise specified

### First Round

The first-round survey identified a total of 446 research priorities. Responses included statements and questions with varying levels of description and detail. These responses were aggregated into 75 unique research priorities. Of these priorities, 23, which were identified more than three times by respondents, were carried forward to the second round. A detailed description of the identified research priorities in round one is presented in Table 2.

**Table 2:** Top research priorities identified in the first round

	Research priority	Frequency reported^a^
1	*Optimal exercise modality*	34
	Which form of preoperative exercise is most beneficial for individuals, including a comparison between HIIT, HIRT, resistance, strength, and cardiovascular training	
2	*Effect of prehabilitation on surgical outcomes*	31
	This includes, postoperative complications (Clavien-Dindo, infections etc.), < 30-day mortality, length of hospital stay, length of ICU stay, and readmissions	
3	*Identifying populations most likely to benefit from prehabilitation*	28
	Determining which group of individuals, considering demographic factors such as age, weight, sex, preoperative fitness, and frailty, respond to prehabilitation	
4	*Optimal nutritional regime*	26
	Identifying the role of, and impact of, optimising nutrition with a dedicated dietician service preoperatively. This includes supplementation (e.g., protein, immunonutrition), and optimal feeding routes (parental, enteral)	
5	*Cost effectiveness of prehabilitation programmes*	23
	This includes a cost benefit analysis of interventions, and establishing what resources are required to set up a prehabilitation programme	
6	*Modes of delivery for prehabilitation*	23
	This includes telehealth programmes, community-based programmes, centre-based programmes, and home programmes in rural and metropolitan settings	
7	*Defining prehabilitation core outcome measures*	19
	To determine a core (minimum) set of outcomes that can be used universally in clinical practice/research to gauge and validate the effect of preoperative interventions	
8	*Optimal psychological interventions*	16
	To examine the role and benefits of preoperative psychological therapies and discussions with a psychologist preoperatively	
9	*Medical optimisation*	15
	The includes management of preoperative iron deficiency, smoking cessation, and alcohol cessation	
10	*Enhancing compliance and adherence*	14
	Establish compliance rates for current prehabilitation programmes and investigate ways to improve compliance, engagement, and adherence	
11	*Optimal duration of prehabilitation programmes*	14
	What is the optimum duration and timing of a prehabilitation programme and does this vary across different tumour streams	
12	Optimal composition of prehabilitation programmes	10
	What is the benefit of a multimodal approach to prehabilitation (combining nutrition, physical fitness, psychosocial wellbeing, and medical optimisation) and what resources should be dedicated to each	
13	*Effect of prehabilitation on patient-reported outcomes*	10
	What is the impact prehabilitation has on patient-reported quality of life, well-being, PROMs	
14	*Screening tools to identify patients for consideration of prehabilitation*	10
	Developing a screening tool to identify frail and at-risk patients for consideration of prehabilitation. This includes using advanced technologies such as AI	
15	*Effect of prehabilitation on functional outcomes*	9
	Exploring the impact of prehabilitation on activities of daily living, mobility, and exercise capacity postoperatively	
16	*Delaying surgery for prehabilitation*	8
	Determining the costs or benefits of delaying surgery to engage in prehabilitation	
17	*Prehabilitation during neoadjuvant therapies*	8
	Can prehabilitation be safely undertaken during neoadjuvant chemotherapy, investigating patient engagement and potential benefits	
18	*Patient education*	6
	Examining the role of patient education in prehabilitation	
19	*Effect of prehabilitation on survival*	6
	This includes overall survival and interval survival rates	
20	*Wearable technology in prehabilitation*	6
	Examining the role of wearable technology in prehabilitation to monitor performance and helping to tailor specific interventions	
21	*Preoperative weight loss*	6
	Does modifying preoperative obesity lead to better postsurgical outcomes	
22	*Effect of prehabilitation on disease recurrence*	4
	Does prehabilitation reduce the rate of oncological recurrence	
23	*Biomarkers and response to prehabilitation*	4
	Determining the relationship between biomarkers and their ability to predict responders to prehabilitation	

^a^Results have been ranked by frequency; when surveys were distributed, these priorities were randomised

*HIIT* high-intensity interval training, *HIRT* high-intensity resistance training, *ICU* intensive care unit, *PROMs* patient-reported outcome measures, *AI* artificial intelligence

### Second Round

Of the 23 research priorities disseminated in round two, 12 reached consensus (> 70% of scores ‘high or very high research priority’). The highest median score was 3, indicating that most identified research priorities carried at least moderate importance. Based on participants’ open feedback, no further changes had to be made to the priorities or descriptions, and there was no addition of priorities by experts. A detailed description of the identified research priorities in round two is presented in Table 3.

**Table 3:** Delphi round two and three results^a^

Top research priorities^b^		Round 2 [*n *= 126]		Round 3 [*n* = 118]	
		Rated as high or very high priority (%)	Median score (IQR)	Rated as high or very high priority (%)	Median score (IQR)
2	*Effect of prehabilitation on surgical outcomes*^*c*^	109 (87)	1 (1–2)	**113 (96)**^**c**^	**1 (1–2)**
	This includes, postoperative complications (Clavien–Dindo, infections etc.), <30-day mortality, length of hospital stay, length of ICU stay and readmissions				
3	*Identifying populations most likely to benefit from prehabilitation*^*c*^	111 (88)	2 (1–3)	**106 (90)**^**c**^	**1 (1**–**2)**
	Determining which group of individuals, considering demographic factors such as age, weight, sex, preoperative fitness, and frailty, respond to prehabilitation				
12	*Optimal composition of prehabilitation programmes*^*c*^	101 (80)	2 (1–2)	**104 (88)**^**c**^	**1 (1**–**2)**
	What is the benefit of a multimodal approach to prehabilitation (combining nutrition, physical fitness, psychosocial wellbeing, and medical optimisation) and what resources should be dedicated to each				
7	*Defining prehabilitation core outcome measures*^*c*^	103 (8)	1 (1–2)	**102 (86)**^**c**^	**2 (1**–**2)**
	To determine a core (minimum) set of outcomes that can be used universally in clinical practice/research to gauge and validate the effect of preoperative interventions				
15	*Effect of prehabilitation on functional outcomes*^*c*^	94 (75)	2 (1–3)	**95 (81)**^**c**^	**2 (1**–**2)**
	Exploring the impact of prehabilitation on activities of daily living, mobility, and exercise capacity postoperatively				
13	*Effect of prehabilitation on patient-reported outcomes*^*c*^	95 (75)	2 (1–3)	**93 (79)**^**c**^	**1.5 (1**–**2)**
	What is the impact prehabilitation has on patient-reported quality of life, well-being, PROMs				
5	*Cost effectiveness of prehabilitation programmes*^*c*^	92 (73)	2 (1–3)	**91 (77)**^**c**^	**2 (1**–**2)**
	This includes a cost benefit analysis of interventions and establishing what resources are required to set up a prehabilitation programme				
10	*Enhancing compliance and adherence*^*c*^	88 (70)	2 (1.25–3)	**89 (75)**^**c**^	**2 (1**–**2)**
	Establish compliance rates for current prehabilitation programmes and investigate ways to improve compliance, engagement, and adherence				
17	*Prehabilitation during neoadjuvant therapies*^*c*^	93 (73)	2 (1–3)	**83 (70)**^**c**^	**2 (1**–**3)**
	Can prehabilitation be safely undertaken during neoadjuvant chemotherapy, investigating patient engagement and potential benefits				
6	*Modes of delivery for prehabilitation*^*c*^	89 (71)	2 (1–2)	**82 (70)**^**c**^	**2 (1.25**–**3)**
	This includes telehealth programmes, community-based programmes, centre-based programmes, and home programmes in rural and metropolitan settings				
14	*Screening tools to identify patients for consideration of prehabilitation*	90 (71)	2 (1–2.75)	81 (69)	2 (1–3)
	Developing a screening tool to identify frail and at-risk patients for consideration of prehabilitation. This includes using advanced technologies such as AI				
4	*Optimal nutritional regimen*	92 (73)	2 (1–3)	80 (68)	2 (1–3)
	Identifying the role of, and impact of, optimising nutrition with a dedicated dietician service preoperatively. This includes supplementation (e.g., protein, immunonutrition) and optimal feeding routes (parental, enteral)				
1	*Optimal exercise modality*	87 (69)	2 (2–3)	–	–
	Which form of preoperative exercise is most beneficial for individuals, including a comparison between HIIT, HIRT, resistance, strength, and cardiovascular training				
11	*Optimal duration of prehabilitation programmes*	87 (69)	2 (1–2)	–	–
	What is the optimum duration and timing of a prehabilitation programme and does this vary across different tumour streams				
16	*Delaying surgery for prehabilitation*	82 (65)	2 (1–3)	–	–
	Determining the costs or benefits of delaying surgery to engage in prehabilitation				
18	*Patient education*	73 (58)	2 (2–3)	–	–
	Examining the role of patient education in prehabilitation				
19	*Effect of prehabilitation on survival*	73 (58)	2 (1.25–3)	–	–
	This includes overall survival and interval survival rates				
9	*Medical optimisation*	72 (57)	2 (2–3)	–	–
	The includes management of preoperative iron deficiency, smoking cessation, and alcohol cessation				
8	*Optimal psychological interventions*	63 (50)	2.50 (2–3)	–	–
	To examine the role and benefits of preoperative psychological therapies and discussions with a psychologist preoperatively				
20	*Wearable technology in prehabilitation*	57 (45)	3 (2–3)	–	–
	Examining the role of wearable technology in prehabilitation to monitor performance and helping to tailor specific interventions				
23	*Biomarkers and response to prehabilitation*	56 (44)	3 (2–3)	–	–
	Determining the relationship between biomarkers and their ability to predict responders to prehabilitation				
22	*Effect of prehabilitation on disease recurrence*	53 (42)	3 (2–4)	–	–
	Does prehabilitation reduce the rate of oncological recurrence				
21	*Preoperative weight loss*	46 (37)	3 (2–3)	–	–
	Does modifying preoperative obesity lead to better postsurgical outcomes				

Bold values represent those which reached concensus definition across round 2 and 3, and represent the top research priorities in pehabilitation for patient undergoing cancer surgery

^a^Likert scale (1 = very high; 2 = high; 3 = moderate; 4 = low; or 5 = very low research priority)

^b^Priorities are listed in order of ‘number of ratings 1 or 2, very high or high research priority’ in round 3

^c^Research priority reached consensus

*IQR* interquartile range, *ICU* intensive care unit, *PROMs* patient-reported outcome measures, *AI* artificial intelligence, *HIIT* high-intensity interval training, *HIRT* high-intensity resistance training

### Third Round

The focused list of 12 research priorities was presented to experts in the third round. A total of 10 prehabilitation research priorities reached consensus (> 70% rated as ‘high or very high research priority’) and formed the final list of top research priorities. The ‘effect of prehabilitation on surgical outcomes’ (median score = 1), ‘identifying populations most likely to benefit from prehabiliation’ (median score = 1) and ‘optimal composition of prehabilitation programmes’ (median score = 1) were research priorities that achieved the highest degree of consensus among prehabilitation experts. The complete list including the top research priorities is presented in Table 3.

## Discussion

To the best of our knowledge, this international Delphi study is the first to identify research priorities in prehabilitation for patients undergoing cancer surgery. We were successfully able to employ a Delphi methodology through an online platform, achieving consensus across a multidisciplinary international group. Of the 446 research priorities identified by a comprehensive group of prehabilitation experts in the first round, 10 achieved consensus and formed the top prehabilitation research priorities for patients undergoing cancer surgery.

The priorities identified in this study address the limitations outlined in the current literature. Recent systematic reviews indicate that prehabilitation has positive effects on reducing hospital length of stay, improving functional recovery, and reducing surgical complications.^1,19^ However much of this evidence is described as low quality, with a heterogenicity across outcome measures, populations and prehabilitation interventions.^9,10^ Prehabilitation experts identified ‘optimal composition of prehabilitation programmes’, ‘identifying populations most likely to benefit from prehabilitation’ and ‘effect of prehabilitation on surgical outcomes’ as important research priorities. Future research in these areas would improve the overall quality of evidence available, assisting in establishing effective prehabilitation programmes that positively impact targeted individuals*.* As prehabilitation gathers further evidence, implementation factors must also be considered. Cost remains one of the major barriers for the adoption of new healthcare systems. This theme was considered important by experts, ‘cost effectiveness of prehabilitation programmes’, and while there are proposed models for evaluating the cost effectiveness of prehabilitation programmes,^20,21^ current literature is lacking. Other priorities identified, including ‘modes of delivery for prehabilitation’ and ‘prehabilitation during neoadjuvant therapies’, aligned with current trends in cancer surgery. For example, telehealth, which gained popularity as a treatment modality, especially following the covid pandemic,^22,23^ represents a vital technological tool in the delivery of healthcare. Additionally, as the implementation of neoadjuvant oncological therapy in pathologies, such as rectal and oesophageal cancer, expands,^24,25^ the safety of prehabilitation during this treatment will require evaluation.

During the second survey round, it was noted that all initially identified priorities received a median score of below 3, indicating that these priorities carried at least moderate importance. However, not all these priorities met the consensus definition. In some of these cases, such as ‘medical optimisation’, ‘optimal exercise modality’ and ‘preoperative weight loss’, this may have been in part due to the existence of high-quality evidence surrounding topics such as weight loss, smoking cessation and preoperative exercise.^26–28^ Research priorities, including ‘optimal exercise modality’ and ‘optimal duration of prehabilitation programmes’ did not meet consensus definition by small margins. Given these priorities reached a practically similar level of agreement and the evidence base for preoperative excerise,^28^ they should also be regarded as important areas of research.

This study was able to recruit a diverse population of experts spanning academic, medical, nursing, and allied health staff. As prehabilitation relies on a synergistic approach from multidisciplinary surgical team members,^9^ their contribution to this study enhanced the validity of our results. However, the process of optimising patients for oncological resections is tailored to specific surgeries/tumour streams. For example, preoperative cardiopulmonary exercise training reduces thoracic complications and length of stay for patients undergoing thoracic resections,^29^ while specific shoulder range-of-motion interventions improve upper limb recovery following mastectomy. Our study encompassed experts in different tumour streams (i.e., colorectal, pancreatic, urological, lung, hepatic, etc.), and remained broad in its definition of cancer surgery. While this may reduce the generalisability of results, it can also be considered a strength; encompassing a wide range of experts allows conclusions to be applied across various oncological specialities.

Further strengths of this study lie in its methodology, as up-to-date guidelines on the reporting of Delphi studies were used to inform our technique, including CREDES and COMET recommendations.^15,16^ Anonymity between experts and results was maintained during each round, which reduced response and participant bias. While there is no defined ideal consensus measurement for Delphi studies, our choice of 70% of responses as ‘very high’ or ‘high’ research priority aligns with current literature for accepted levels of agreement.^30^ The online nature of this study allowed the inclusion of international participants across four continents, which enhances the validity of results. An additional advantage of our online platform was the relative speed of data collection, ensuring relevancy of our results in the context of ever-emerging evidence. As many responses to our initial open-ended question were collated to 75 research priorities, we are confident that we included the top research priorities in our analysis.

As prehabilitation remains a field with great interest and ever-evolving research, we anticipate that research priorities for patients undergoing cancer surgery will change with time. We suggest that a new Delphi study be completed within the next 5–10 years to aid guiding future research. As more information is gathered, we anticipate that certain priorities identified in this study will become more specific, while new priorities may also emerge.

### Limitations

A limitation of many Delphi studies is engagement. Our response rate of 29% can be considered low but is in keeping with other literature with similar methodology.^31–33^ Although an increased response rate would improve the validity of results, it is unclear what impact it would have on the final priority list given the recurrence of many responses to our initial open-ended question. Our initial open-ended question also received responses with significantly variable levels of detail. Responses included statements and questions, encompassing both very broad and very specific research priorities. Very detailed responses were encompassed into broader research topics, which may have resulted in a loss of detail or an important research priority. We limited this effect this by carrying out the collation through discussion between a group of four experts, ensuring that descriptions adequately represented the constituent responses. Our study used various techniques, including individual reminders and short surveys, to enhance participation in each round (86%, 76% and 72% in the first, second and third rounds, respectively). However, this could be improved to ensure representativeness of results to our cohort. A disadvantage of the online platform is the inability to engage in collaborative discussion with participants regarding rationale of their responses, which is a hallmark of the Delphi technique. Although open-ended responses were invited in rounds two and three, the authors note that very few participants gave meaningful feedback. This could be improved by employing a face-to-face component of priority setting, such as the James Lind Alliance^34^ methodology. Purposive and snowball sampling were used to recruit participants for this study, which means we cannot be sure that participants are truly representative of prehabilitation experts globally. Cohort or other random sampling methods could be used to improve this. Our study focused on the opinion of prehabilitation experts; the opinions of patients and carers were not incorporated but would have contributed positively to our agenda. We suggest that future studies aim to incorporate the perspective of patients, as prehabilitation is patient centric and tailored to individual needs; these findings may be used to facilitate this arm of research. The identified research priorities can also be used to support specific inquiries into different tumour streams, e.g. colorectal, to identify the most important prehabilitation research priorities within them.

## Conclusion

We were able to successfully implement an international Delphi study that established consensus on the top 10 research priorities in the field of prehabilitation for patients undergoing cancer surgery. This promotes new evidence in the field of prehabilitation, which has been shown to have significant benefits to patients. These priorities will be useful to clinicians and researchers to efficiently focus academic efforts and resources on areas of high importance.
